# Functional connectivity of hippocampal subregions in PTSD: relations with symptoms

**DOI:** 10.1186/s12888-018-1716-9

**Published:** 2018-05-15

**Authors:** Bailee L. Malivoire, Todd A. Girard, Ronak Patel, Candice M. Monson

**Affiliations:** 1 Department of Psychology, Ryerson University, 350 Victoria St, Toronto, ON M5B 2K3 Canada; 2 Department of Clinical Health Psychology, University of Manitoba, Winnipeg, MB Canada

**Keywords:** Posttraumatic stress disorder, Functional magnetic resonance imaging, Hippocampus, Trauma symptoms, Resting state connectivity

## Abstract

**Background:**

Posttraumatic stress disorder (PTSD) is associated with abnormal hippocampal activity; however, the functional connectivity (FC) of the hippocampus with other brain regions in PTSD and its relations with symptoms warrants further attention. We investigated subregional hippocampal FC in PTSD during a resting state compared with a trauma-exposed control (TEC) group. Based on extant research, we targeted the FCs of the anterior and posterior hippocampal subregions with the amygdala, medial prefrontal cortex (mPFC), and the posterior cingulate (PCC).

**Methods:**

Resting-state functional magnetic resonance images were acquired from 11 individuals with PTSD and 13 trauma-exposed controls. Anterior and posterior hippocampal FC was compared between groups. Within the PTSD and TEC groups, subregional hippocampal FC was correlated with scores on the Clinician-Administered PTSD Scale (CAPS) at time of scan and 4 months post-scan.

**Results:**

Those with PTSD had significantly greater FC compared with the TEC group between the left posterior hippocampus and the bilateral PCC (*g*’s > .96). Direct contrasts of the Fisher z-transformed coefficients indicated that the correlations between CAPS scores 4 months post scan and the FC between the left hippocampal head and the right PCC (z = − 2.07, *p* = .039) as well as the FC between the right hippocampal tail and the right mPFC (z = − 2.19, *p* = .029) were significantly greater in the PTSD group compared to the TEC group.

**Conclusions:**

These results support between-group differences in posterior hippocampal FC and different relations with PTSD future symptoms, underscoring associations with the anterior and posterior hippocampus. These findings enrich our understanding of PTSD pathophysiology and provide support for future investigations of imaging biomarkers predictive of disease progression.

## Background

Approximately 10% of individuals exposed to a traumatic event go on to be diagnosed with posttraumatic stress disorder (PTSD), a debilitating psychiatric condition [1]. Neural differences have been noted among those with PTSD compared with those exposed to trauma but not diagnosed with the disorder [2, 3]. According to the widely-adopted neurocircuitry model, PTSD is primarily associated with structural and functional changes in the amygdala, medial prefrontal cortex (mPFC), and hippocampus [4, 5]. Investigation of neural correlates that are associated with greater likelihood of developing PTSD following trauma exposure enriches our understanding of disorder biomarkers and could guide therapeutic targets [6, 7]. The primary objective of the present study was to investigate subregional hippocampal neural correlates of current and future symptoms in PTSD.

Current theories of PTSD identify hippocampal dysfunction as a key contributor to hallmark symptoms of PTSD including trauma-related intrusions, difficulty voluntarily recollecting trauma details, and overgeneralization of fear [1, 8, 9]. Moreover, individuals with PTSD demonstrate difficulties with everyday memory [10], working memory [11], and episodic memory [12, 13]. Given the integral role of the hippocampus in PTSD symptoms, memory, and related processes, it is of interest to understand how the role of the hippocampus in the neurocircuitry model differs between individuals with PTSD and a trauma-exposed control (TEC) group, and the functional connectivity within this system to PTSD symptoms.

Resting-state functional connectivity (rsFC) is commonly used to explore patterns of brain system activity in psychiatric conditions, such as PTSD [7, 14]. rsFC is a powerful tool that assesses correlated activity across brain regions over time during rest, permitting investigation of functional networks independent of a task to characterize abnormal intrinsic brain activity [7]. Thus, rsFC is a sensitive imaging technique that offers potential to identify imaging biomarkers of PTSD. rsFC studies in PTSD have found group differences in FC using whole-brain analyses [15], as well as using seed region-of-interest (ROI) analyses [16–18]. For instance, rsFC differences in hippocampal FC between PTSD and controls have been observed based on analyses using the amygdala [17, 18], the PCC [16], and the mPFC [19] as seed regions. However, functional magnetic resonance imaging (fMRI) research has largely focused on the hippocampus as a unitary structure. Given the different structural and functional connectivities of hippocampal subregions [20–22], investigating hippocampal rsFC at a subregional level may further elucidate the functional neurobiology of PTSD.

Anterior and posterior hippocampal subregions have distinct structural and functional connections and contribute to different emotional and cognitive processes (see [23] for a review). For example, the posterior hippocampus has connections with the pregenual ACC, the PCC, and the precuneus [21], and makes a greater contribution to the default mode network compared with the anterior hippocampus [20]. The posterior hippocampus specifically plays an important role in memory retrieval and spatial cognition [24]. In contrast, the anterior hippocampus is preferentially associated with the amygdala [22], the hypothalamic-pituitary-adrenal (HPA) axis, and the limbic prefrontal circuitry compared with the posterior hippocampus [20, 25]. Neuroimaging studies have linked anterior hippocampal activation with emotional memory [26] and reward-directed behaviour [27]. Anterior hippocampal-amygdala connections are thought to underlie atypical memory processes in PTSD, including flashbacks, intrusive thoughts, and nightmares [9], as well as overgeneralization of fear [28]. In sum, there is evidence to support differential roles of the anterior and posterior hippocampal subregions in memory processes, and PTSD symptoms and associated processes. Thus, here we investigated differential FC with these subregions to provide added insight into hippocampal FC in PTSD compared with TEC.

Limited prior research has investigated subregional hippocampal rsFC in PTSD. Chen and Etkin [20] compared anterior and posterior hippocampal rsFC between patients with Generalized Anxiety Disorder, PTSD, and non-traumatized controls. Compared with both the anxiety and control groups, connectivity to the PTSD group was diminished between the posterior hippocampus and brain regions part of the default mode network (associated with self-related cognitive processes) [29, 30], including the PCC and pregenual ACC. The PTSD and GAD groups differed from controls in their anterior hippocampal connectivity to the dorsal ACC/pre-supplementary motor area. Chen and Etkin [20] acknowledged the lack of a TEC group as a limitation: a TEC group is important to elucidating abnormal connectivity differences among individuals exposed to trauma who develop PTSD compared with those who do not. A recent study by Lazarov, Zhu, Suarez-Jimenez, Rutherford, and Neria [31] compared rsFC of the anterior and posterior hippocampus with brain regions within the salience and default-mode networks in PTSD patients and TECs. No between-group differences were evident when the hippocampus was treated as a homogenous structure. However, when investigating the anterior and posterior subregions separately, decreased rsFC was observed between the anterior hippocampus and the precuneus within the PTSD group compared with TEC. These recent studies [20, 31] highlight the importance of investigating subregional hippocampal rsFC.

rsFC can be used to predict PTSD symptoms measured by the Clinician Administered PTSD Scale (CAPS), a well-validated measure of PTSD symptom severity [32]. For instance, PCC/precuneus connectivity with the amygdala in PTSD patients is positively associated with CAPS scores and predictive of future symptoms 6 weeks later [33]. PCC connectivity with the right hippocampus, right amygdala, left mPFC, and left superior temporal gyrus are negatively correlated with CAPS scores [19, 34]. Moreover, the strength of the connectivity between the PCC and amygdala predicted PTSD symptom severity 1 to 6 months following the traumatic event [19]. Investigating relationships between FC and PTSD symptom severity can help identify possible biomarkers of PTSD or identify individuals with a greater predisposition for the disorder [19]. The hippocampus is a central structure in the neurocircuitry model of PTSD [4] and abnormal hippocampal connectivity is proposed to underlie key symptoms in PTSD [9]. Thus, subregional hippocampal rsFC may be predictive of PTSD symptom severity and could be a biomarker of the disorder; however this has not previously been investigated directly.

In sum, current theories of PTSD identify hippocampal dysfunction as a key contributor to hallmark symptoms of PTSD [1, 8, 9]. To our knowledge, only one study [31] has used subregional hippocampal seed regions to compare rsFC between PTSD and TEC groups. This level of analysis provides a more nuanced understanding of hippocampal connectivity. Moreover, given the integral role of the hippocampus in PTSD symptoms, subregional hippocampal rsFC is a likely predictor of PTSD symptom severity; however, no studies to date have investigated this association. The present study first compared subregional hippocampal rsFC with brain regions part of the neurocircuitry model between PTSD and TEC groups. A priori hypotheses were made based on previous rsFC research in PTSD and neuroanatomical connectivity. It was hypothesized that the PTSD and TEC groups would differ in rsFC between the anterior hippocampus and the amygdala and mPFC and between the posterior hippocampus and PCC. Secondly, the present study builds on existing literature by investigating the associations between subregional hippocampal rsFC and current PTSD symptoms, as well as the ability of subregional hippocampal rsFC to predict future symptoms. Given that the anterior and posterior hippocampal subregions contribute to different emotional and cognitive processes it was expected that these regions would differentially predict PTSD symptoms. Based on the literature reviewed above, we predicted that greater functional connectivity of the anterior hippocampus and lower connectivity of the posterior hippocampus would predict greater symptom severity.

## Method

### Participants

The current rsFC study sample (*N* = 24) comprised participants participating in larger studies of recently traumatized individuals; task-related fMRI data are reported by Patel, Girard, Pukay-Martin, and Monson [35]. Participant demographics and clinical characteristics are summarized in Table 1. At the time of scan, 11 participants met criteria for PTSD and the remaining 13 for the TEC group as determined by both CAPS and a psychodiagnostic assessment. CAPS has been demonstrated to have excellent reliability and validity for assessment of PTSD symptoms [32]. A CAPS score greater than 45 was required for a diagnosis of PTSD. In addition, consistent with Diagnostic and Statistical Manual of Mental Disorders, 4th Edition, (DSM-IV) criteria, the participants needed to endorse the presence of at least one re-experiencing symptom, three numbing or avoidance symptoms, and two hyperarousal symptoms. Diagnostic thresholds for these items were scores of at least one on frequency and two on intensity within the past month. CAPS was administered at the time of scan and 4 months post scan. The PTSD group was exposed to a variety of traumatic events including sexual assault (*n* = 5), physical assault and/or death threats (*n* = 4), witnessing a violent physical assault or death (*n* = 1), and combat exposure (*n* = 1).

**Table 1:** Participant demographics and clinical characteristics

Group	TEC (*n* = 13)	PTSD (*n* = 11)	*p*	*d*
Sex (male:female)	4:9	5:6	.459	0.31
Age	32 (10.4)	34 (13.6)	.623	0.17
Education	14.5 (2.1)	14.0 (2.5)	.560	−0.22
CAPS at scan	12.5 (10.0)	70.0 (14.2)	< .001	4.68
CAPS 4-mo post-scan	8.9 (16.5)	48.3 (26.2)	.002	1.80
BDI-II	6.0 (8.3)	18.9 (12.5)	.010	1.22
Time since trauma (mo)	10.0 (4–24)	12.0 (3–372)	.296	0.44

Sex differences are reported as frequency (*n*) and were evaluated with a Chi-square contingency test, Time since trauma is reported as medians (range) and compared with a Mann-Whitney U test (due to non-normality), and the remaining demographics are reported as Means (*SD*) using independent t tests. All contrasts were converted to Cohen’s *d* to assess effect size

*Abbreviations*: *BDI* Beck Depression Inventory, *CAPS* Clinician Administered PTSD Scale, *mo* months, *PTSD* posttraumatic stress disorder, *TEC* trauma-exposed controls

The requirements for the TEC group were exposure to a traumatic event in accordance with criterion A of the CAPS and that they had a total CAPS score of less than 30 at time of scan. A cut-off score of 30 was selected to ensure the TEC group was diagnostically distinct from the PTSD group. The types of trauma exposure varied within the TEC group and included motor vehicle or biking accident (*n* = 5), witnessing a violent assault and/or death threats (*n* = 4), and direct exposure to physical assault or death (*n* = 4). As expected, the PTSD group had significantly higher CAPS scores at the time of scan and 4 months post-scan compared with the TEC group. The groups did not differ significantly in sex, age, education, or time since trauma (see Table 1).

Psychological comorbidities were assessed using either the Mini International Neuropsychiatric Interview (*n* = 18) [36] or the Structured Clinical Interview for DSM-IV (*n* = 6) [37] depending on study recruitment. Comorbid diagnoses within the PTSD group included anxiety (*n* = 6), major depressive (*n* = 4), and substance use (*n* = 2) disorders. Five PTSD participants were taking antidepressant medications at the time of assessment. With regard to the TEC group, only two participants were diagnosed with an anxiety disorder, and one had both alcohol abuse and substance dependence diagnoses; two participants were taking antidepressant medication. The Beck Depression Inventory-II (BDI-II) [38] was also used to assess depressive symptoms. As anticipated, compared with the TEC group, the PTSD group had significantly higher depression scores (see Table 1).

### Brain image acquisition and preprocessing

Anatomical and resting-state fMRI images were acquired in an axial orientation using a 3-T Sigma MR System (GE Medical Systems, Milwaukee) located at Toronto Western Hospital. T_1_-weighted anatomical scans were acquired using a fast spoiled gradient echo (FSPGR) sequence at a resolution of isotropic 1-mm^3^ voxels (176 slices, 256-mm FOV, 256 × 256 matrix). As outlined below, these T_1_ scans were used to co-register the resting-state functional scans, derive tissue-segmented maps, and determine normalization parameters. Resting-state data were collected for 308 s (TR = 2000 ms, TE = 30 ms, flip angle = 85°, 40 slices) at a spatial resolution of 3.125 × 3.125 × 4.0 mm^3^ voxels (FOV = 200 mm). During the resting-state scan, participants laid face upward with their eyes open while viewing a black fixation cross on a white screen for 5 min. The resting state scans were preprocessed using the Data Processing & Analysis of Brain Imaging (DPABI) toolbox [39], which includes Data Processing Assistant for Resting-State fMRI Advanced (DPARSFAv3.2) and was run on MATLAB version 7.10.0 (The MathWorks, Natick, MA). Each participant’s resting state scan consisted of 154 time points. The first 10 time points were removed for each participant for signal equilibrium and the remaining 144 images were preprocessed. The images were corrected for slice timing and a Friston 24-parameter head motion correction was applied, which regresses out head motion effects from the realigned images [40]. In addition, nuisance covariates including white matter and cerebrospinal fluid were regressed. White matter and cerebrospinal fluid masks were generated from each participant’s segmentation map using a probability threshold of 0.99. The nuisance covariates were regressed from the time series using the CompCor method [41], which creates a combined white matter/cerebrospinal fluid mask and extracts the first five principal components to reduce noise related to respiratory and cardiac effects. Time points with too much motion were defined as volumes with FD (Jenkinson) > 0.2 mm and volumes two forward one back from these volumes [42], which is consistent with recommended DPABI methods [39]. Times points with motion above this threshold were regressed out.

The DARTEL tool [43] was used to normalize the functional images into a standard stereotaxic anatomical Montreal Neurological Institute (MNI) space. The normalized volumes were resampled to a voxel size of 3 mm^3^. This process involved three steps including coregistration, segmentation, and writing normalization parameters. The images were smoothed with a 4-mm full-width, half-maximum isotropic Gaussian kernel, as in similar reports in the PTSD rsFC literature (e.g., [19]). Temporal filtering (0.01–0.1 Hz) was applied following smoothing to the time series of each voxel to reduce the effect of low and high frequency noise. Using the quality control feature of DPABI the accuracy of the coregistration, segmentation, and normalization of the images were checked. The head motion metrics were also reviewed for each participant. Lastly, a mean regression and standard deviation division standardization module was applied, in accord with recommendations to reduce the impact of nuisance variation on resting state fMRI measures [39].

**Fig. 1:**
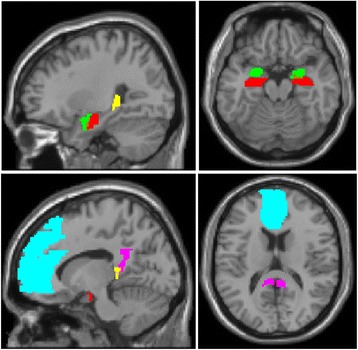
The ROIs used in the present study displayed on a template brain. Red = anterior hippocampus; yellow = posterior hippocampus; green = amygdala; cyan = mPFC; magenta = PCC

Hypothesis-driven ROI-based analyses were used to directly target the study aims. This approach also mitigates the severity of correction for multiple tests. Based on a priori hypotheses, left and right ROIs for the hippocampus, mPFC, PCC, and amygdala were constructed using Mask for Region of Interest Analysis (MARINA) software [44]. The mPFC ROI was chosen to be comprehensive and included several regions shown to be implicated in PTSD pathophysiology, including the medial frontal gyrus and anterior cingulate [45], and to protect against Type 1 error involved in multiple ROI comparisons. As shown in Fig. 1, the hippocampal masks were manually sectioned into anterior and posterior ROIs using MRIcron [46], based on guidelines for anatomical segmentation of the hippocampal head and tail, respectively [47].

### Data analysis

Spatially averaged time series from each ROI were extracted for each participant and used to generate FC maps using a voxel-wise method. The correlation coefficient map was converted into a Fisher’s *z* map using the Fisher’s *z* transformation to improve normality for analyses. The mean z values representing FC between each pair of ROIs were extracted for analysis. These FC coefficients were correlated with CAPS scores at the time of scan and for a subset for whom symptom data were available 4 months post-scan (TEC, *n* = 10; PTSD, *n* = 6;). Consistent with similar studies (e.g., [17]), we first conducted one-sample t-tests to examine between which regions correlated activity (FC coefficients) differed significantly from zero within the PTSD and TEC groups (*α* = .05). However, to minimize Type 1 error and limit analyses to only meaningful FC relations, we only examined between-group differences when there were significant FC within at least one group.

## Results

### Within-group FCs

As shown in Table 2, many of the hippocampal FCs were significant within groups. More specifically, within the TEC group, the FCs between the left hippocampal head and the bilateral amygdala, bilateral mPFC, and left PCC were significant, as well as the FCs between the right hippocampal head and the amygdala ROIs bilaterally, and between the right hippocampal tail and the right amygdala. However, the left hippocampal tail failed to reveal any significant FCs with the other ROIs. In the PTSD group, FC values were significant between the bilateral hippocampal head and the bilateral amygdala, and between the left hippocampal tail and the bilateral PCC. FC did not differ significantly from zero between the right hippocampal tail and any of the ROIs. Five of the above FCs survived a Bonferroni correction (*p* < .001), with the most robust FCs between the hippocampal head and amygdala ROIs for both groups with medium-large effect sizes (see Table 2).

**Table 2:** Means and standard deviations for the FC coefficients between the hippocampal head and tail with the amygdala, mPFC, and PCC regions

Group	Hippocampus		Amygdala		mPFC		PCC	
			Left	Right	Left	Right	Left	Right
PTSD	Head	Left	.64(.24)***	.57(.25)**	.31(.37)	.24(.41)	.24(.40)	.20(.44)
		Right	.45(.27)**	.58(.22)***	.31(.35)	.28(.37)	.22(.39)	.23(.40)
	Tail	Left	.27(.34)	.24(.37)	.33(.37)	.29(.39)	.33(.35)*	.35(.35)*
		Right	.22(.38)	.19(.39)	.18(.45)	.21(.42)	.21(.39)	.27(.38)
TEC	Head	Left	.64(.14)***	.43(.19)***	.23(.28)*	.18(.27)*	.16(.20)*	.12(.24)
		Right	.39(.22)***	.54(.16)***	.06(.19)	.04(.18)	−.04(.19)	−.01(.25)
	Tail	Left	.05(.23)	.07(.22)	.06(.15)	−.01(.17)	.08(.15)	.08(.20)
		Right	.08(.23)	.15(.23)*	.06(.19)	.04(.19)	.07(.21)	.09(.19)

*p < .05, **p < .01, ***p < .001 for the one-sample t-tests, rsFC > 0

FC values reported in the table are *r* coefficients to aid interpretation; analyses were conducted on the Fisher z-transformed coefficients to meet distribution assumptions

### Between-group contrasts

**Fig. 2:**
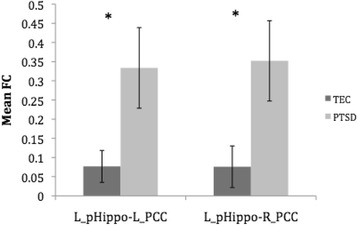
The mean FC *r* values for the PTSD and TEC groups between the left hippocampal tail and the bilateral PCC. Error bars represent standard error

As shown in Table 2, the mean FC values were higher in magnitude in the PTSD group than TEC across all hippocampal-ROI pairs, but they were also more variable. Among the FCs that were significant within groups, the correlated activation between the left hippocampal tail and the left PCC, *t*(22) = 2.166, *p* = .041, *g* = .96, and right PCC, *t*(22) = 2.129, *p* = .045, *g* = .97, was also significantly greater for the PTSD group compared to the TEC group and revealed comparably large effect sizes (Fig. 2).

### Correlations with PTSD symptoms

Correlation analyses following up the two significant between-group FCs of the left hippocampal tail with bilateral PCC revealed that symptom severity 4 months post-scan was significantly predicted by the FC between the hippocampal tail and PCC in the left hemisphere, *r* = −.87, *p* = .026.

**Fig. 3:**
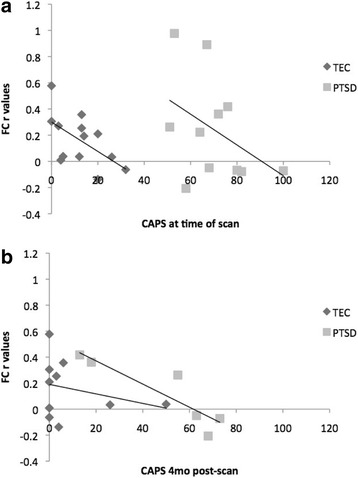
Associations between PTSD symptoms (CAPS) and the FC between the left hippocampal head and the left PCC for the TEC and PTSD groups. There was a significant, negative correlation between (**a**) FC and symptoms for the TEC group at the time of scan (*R*^2^ = .33) and (**b**) for the PTSD group for symptoms 4-months post-scan (*R*^2^ = .80)

Further exploratory analyses between FCs and symptoms revealed that CAPS scores at the time of the scan were negatively associated with the FC between the right hippocampal head and the left (*r* = −.64, *p* = .018) and right mPFC (*r* = −.76, *p* = .003) for the TEC group. CAPS scores at the time of the scan were also negatively associated with the FC between the left hippocampal head and the left (*r* = −.57, *p* = .042; Fig. 3a) and right PCC (*r* = −.64, *p* = .019) for the TEC group. In the PTSD group, the above associations were of medium magnitude but failed to reach significance, *r*s > −.44, *p*s > .181.

CAPS scores 4 months post scan were negatively associated with FCs between the right hippocampal head and the right amygdala (*r* = −.69, *p* = .029) for the TEC group. This association was positive, but not significant within the PTSD group, *r* = .27, *p* = .540. Rather, within the PTSD group, CAPS scores 4 months post scan were negatively associated with FCs between the left hippocampal head and the left (*r* = −.90, *p* = .014; Fig. 3b) and right PCC, *r* = −.87, *p* = .016. Symptom severity 4 months post-scan was also significantly associated with FC between the right hippocampal tail and the right mPFC (*r* = −.85, *p* = .031) within the PTSD group. The aforementioned associations were not significant within the TEC group, *r*s ranged from −.40 to .24, *p*s > .255. Direct contrasts of the Fisher z-transformed coefficients further indicated that the correlations between CAPS scores 4 months post scan and the FC between the left hippocampal head and the right PCC (*z* = − 2.07, *p* = .039), as well as the FC between the right hippocampal tail and the right mPFC (*z* = − 2.19, *p* = .029), were significantly greater in the PTSD than TEC group. The other tests of the correlations by group failed to reach significance.

## Discussion

The present study provides empirical support for distinct rsFC among hippocampal subregions when investigating PTSD pathophysiology and its relation with PTSD symptoms. Comparing rsFC between groups we found greater rsFC between the posterior hippocampus and bilateral PCC in the PTSD group compared to the TEC group, which may reflect a biomarker of PTSD pathophysiology or of resilience in the TEC group. This relation on the left was also predictive of symptom severity 4 months post-scan.

Further analyses revealed lower FC between the anterior hippocampus and the bilateral PCC and FC between the posterior hippocampus and the left PCC and right mPFC in the PTSD sample that was associated with greater symptoms 4 months following the resting-state scan. In the TEC group, lower FC between the anterior hippocampus and the bilateral mPFC and PCC were associated with greater current PTSD symptom severity and lower FC between the anterior hippocampus and the right amygdala was associated with higher symptoms 4 months later. Notably, the left-head – right PCC and right-tail – right-mFPC predictors of CAPS scores 4 months post scan were also significantly greater in the PTSD than TEC group. These findings may reflect meaningful differential relations to symptoms reflective of pathophysiology. However, caution is warranted given their exploratory nature and the observation of greater variability and mean FCs in the PTSD group that may reflect psychometric sensitivity. Regardless, these relations warrant further investigation.

The correlation analyses extend previous research by demonstrating that PTSD symptoms were preferentially associated with anterior hippocampal rsFC. This finding is consistent with research that suggests the anterior hippocampus plays a greater role in PTSD symptoms and associated processes compared to the posterior hippocampus. Specifically, anterior hippocampal-amygdala FC is hypothesized to underlie the hallmark intrusion symptoms in PTSD, including intrusive thoughts, flashbacks, and nightmares [9], as well as overgeneralization of fear [28]. Moreover, anterior hippocampal FC is preferentially involved in emotional memory [26] and reward-directed behaviour [27], which may contribute to avoidance, a maladaptive behaviour that maintains PTSD symptoms. Specifically, it is proposed that following trauma exposure individuals with PTSD overconsolidate the traumatic memory, which is easily reactivated by trauma reminders and accompanied by conditioned emotional arousal. This leads to hypervigilance towards trauma-related cues and subsequent avoidance of trauma-related memories and reminders to minimize associated distress [48]. The avoidance could be conceptualized as a form of negative reinforcement that is rewarding for individuals with PTSD. Future research is warranted to investigate anterior hippocampal FC as a predictor of PTSD symptoms and its relationship with avoidance.

With respect to the group contrasts, the PTSD group revealed greater FC between the posterior hippocampus and PCC compared to TEC participants. Posterior hippocampal FC with the cingulate, precuneus, and visual cortices has been linked to spatial processing, including spatial memory and navigation [22]. There is preliminary support for greater impairment in PTSD compared to TEC samples on visuospatial copying tasks [49] and in constructing a cognitive map of a virtual environment [50]. The abnormal FC between the posterior hippocampus and PCC in PTSD compared to the TEC group may reflect differences in these processes and is a future direction for task-based imaging research. Moreover, the PCC and hippocampus are part of the default mode network, which is associated with self-referential thought processes and autobiographical memory [29]. Lower FC within the default mode network is more frequently observed across psychiatric and neurological conditions, with some variation across its subnetworks [30]. Hippocampal-PCC connectivity is associated with memory functions and the current enhanced FC may reflect a unique role of this subnetwork in PTSD. Previous research that has investigated differences in the default mode network between PTSD and TEC samples also support group differences in this network [16, 17]. Group rsFC differences between the hippocampus and PCC may reflect group differences in integration and contextualization of memories, which may contribute to threat hypersensitivity evident in PTSD [16]. Previous research comparing rsFC between individuals with PTSD and trauma-naïve controls has found there to be less hippocampal-PCC rsFC in the PTSD group compared to controls [16, 20]. This discrepancy from the greater FC observed here may be attributable to differences in data preprocessing, sample characteristics and a trauma-exposed versus trauma-naïve control group. These distinctions highlight the need for more consistent methodology across imaging studies and further assessment of moderating factors in PTSD research. Moreover, research is warranted comparing FC between individuals with PTSD, trauma-naïve controls, and TEC samples to characterize normal trauma-related changes in FC and FC specific to PTSD [3].

Two of the hypothesized group differences in rsFC, those between the anterior hippocampus and the amygdala and the posterior hippocampus and the mPFC, were unsupported in the present study. One possibility is that the subregional hippocampal-amygdala rsFC group differences were too subtle to detect with the current design or they may not have been present within this sample. Consistent with the latter, a previous rsFC study also failed to find hypothesized group differences in hippocampal-amygdala activity [51]. Moreover, as reported in Table 2, the FCs between anterior hippocampal ROIs with the amygdalae had the highest mean FC coefficients within each group and were comparable across groups. This finding may reflect that the functional connectivity between the hippocampal head and amygdala may remain robust and intact in PTSD under resting conditions or that this FC is similarly high in both trauma-exposed samples (PTSD and TEC); future comparison to a non-traumatized group will be valuable to assess these possibilities. Of note, a recent meta-analysis of task-based brain activation, revealed hippocampal and amygdala hyperactivity in PTSD in comparison to trauma-naïve samples but not TEC samples [2]. Interestingly, decreased connectivity between these regions was strongly associated with PTSD symptoms in the current TEC sample.

In contrast to the lack of group differences in hippocampal-mPFC rsFC in the present sample, Jin et al. [15] found weaker connectivity between the mPFC and the hippocampus in their PTSD group compared to trauma-naïve controls. Group FC differences are suggested to be a function of the type of comparison group (i.e., trauma-naïve controls versus TEC) [3]. It is possible that changes in hippocampal-mPFC FC are normal following exposure to trauma-related stress and do not differentiate individuals with PTSD from those exposed to trauma who did not develop PTSD. It is also possible that the mPFC ROI we used was too liberal to detect group differences or that group differences were not present in this sample. These are notable negative findings that require further investigation and replication.

Interpretations of the present study findings should be considered in light of the study’s limitations. Firstly, although the ROIs were selected based on a priori hypotheses and the PTSD imaging literature, the small sample size constrains the positive predictive value of the findings. To address reproducibility concerns, we propose future pre-registration of larger-scale, collaborative replications [52, 53]. In addition, the sample was heterogeneous in the types of trauma exposure and time since trauma. In light of evidence that longer resting-state imaging time frames of 9–13 min may improve the test-retest reliability of the scans and FC estimates [54], future replication studies should consider using longer resting-state durations. Of note, post-hoc assessment of the potential relationship between time since trauma and CAPS scores failed to reveal any relations (data not shown). Future research with larger samples should investigate whether the associations found in this study are potentially moderated by type of trauma, time since trauma, and cumulative trauma load. Nonetheless, the current findings are valuable in demonstrating that there are common FC differences that distinguish PTSD and TEC samples. Consistent with previous studies investigating rsFC in PTSD (e.g., [16, 20]), participants had psychological comorbidities and were using antidepressant medication. It is unclear how medication use and psychological comorbidities affect rsFC. However, it is unlikely that the FC differences observed in the present study were solely attributable to medication use and psychological comorbidities given these confounds were present in both the PTSD and TEC groups. Depression scores significantly differed between groups and thus depression symptoms could be influencing the group differences found in the study; however, recruiting individuals with no depression affects generalizability and statistically covarying for depression can inappropriately remove true-score variance shared with diagnostic status [55]. Nonetheless, future neuroimaging research in PTSD is required to investigate the effects of medication use and comorbid symptoms on rsFC. Given heterogeneity of the mPFC and amygdala, another future direction could be to assess the potential of more nuanced rsFC between subregional hippocampal, amygdalar, and functionally distinct mPFC subregions, and their relations with PTSD symptoms. This work may also yield further insight into different symptom profiles. For instance, Nicholson et al. [56] observed differential rsFC between amygdala subregions among those with and without the dissociative subtype of PTSD.

## Conclusions

This study is the first to investigate associations between anterior and posterior hippocampal rsFC and current and future PTSD symptoms and alludes to the anterior hippocampus as a potentially superior biomarker compared to the posterior hippocampus. The group differences in rsFC between the posterior hippocampus and the PCC may reflect a marker of resilience in individuals exposed to trauma who did not go onto develop the disorder. These findings underscore the importance of considering hippocampal subregions independently when investigating FC. This subregional approach extends our understanding of PTSD pathophysiology and provides support for future investigations of subregional hippocampal FC in PTSD and its use as a prognostic aid.

## Abbreviations

ACC: Anterior cingulate cortex
BDI-II: Beck Depression Inventory-II
CAPS: Clinician-Administered PTSD Scale
DPABI: Data Processing & Analysis of Brain Imaging toolbox
DPARSFA: Data Processing Assistant for Resting-State fMRI Advanced
DSM-IV: Diagnostic and Statistical Manual of Mental Disorders, 4th Edition
FC: Functional connectivity
fMRI: Functional magnetic resonance imaging
FSPGR: Fast spoiled gradient echo
HPA: Hypothalamic-pituitary-adrenal
MARINA: Mask for Region of Interest Analysis
MNI: Montreal Neurological Institute
mPFC: Medial prefrontal cortex
PCC: Posterior cingulate cortex
PTSD: Posttraumatic stress disorder
ROI: Region-of-interest
rsFC: Resting-state functional connectivity
TEC: Trauma-exposed control
